# COVID-19 preclinical models: human angiotensin-converting enzyme 2 transgenic mice

**DOI:** 10.1186/s40246-020-00272-6

**Published:** 2020-06-04

**Authors:** Cathleen Lutz, Leigh Maher, Charles Lee, Wonyoung Kang

**Affiliations:** 1 The Jackson Laboratory, 600 Main Street, Bar Harbor, Maine 04609 USA; 2 The Jackson Laboratory for Genomic Medicine, 10 Discovery Drive, Farmington, CT 06032 USA; 3 Precision Medicine Center, The First Affiliated Hospital of Xi’an Jiaotong University, 277 West Yanta Rd., Xi’an, 710061 Shaanxi, People’s Republic of China

**Keywords:** COVID-19, SARS-CoV-2, Coronavirus, Angiotensin-converting enzyme 2 (ACE2), Transgenic mouse

## Abstract

Coronavirus disease 2019 (COVID-19) is a declared pandemic that is spreading all over the world at a dreadfully fast rate. Severe acute respiratory syndrome coronavirus-2 (SARS-CoV-2), the pathogen of COVID-19, infects the human body using angiotensin-converting enzyme 2 (ACE2) as a receptor identical to the severe acute respiratory syndrome (SARS) pandemic that occurred in 2002–2003. SARS-CoV-2 has a higher binding affinity to human ACE2 than to that of other species. Animal models that mimic the human disease are highly essential to develop therapeutics and vaccines against COVID-19. Here, we review transgenic mice that express human ACE2 in the airway and other epithelia and have shown to develop a rapidly lethal infection after intranasal inoculation with SARS-CoV, the pathogen of SARS. This literature review aims to present the importance of utilizing the human ACE2 transgenic mouse model to better understand the pathogenesis of COVID-19 and develop both therapeutics and vaccines.

## Introduction

Coronavirus (CoV), which belongs to the subfamily Coronavirinae, family Coronavirdiae, and order Nidovirales, has repeatedly crossed species barriers, and some have become important human pathogens [1]. In December 2019, a highly pathogenic novel coronavirus, severe acute respiratory syndrome-associated coronavirus-2 (SARS-CoV-2), emerged as the cause of coronavirus disease 2019 (COVID-19). This virus which likely originated from bats then transmitted into/among humans [1] has affected 212 countries and territories around the world causing over 3.5 million confirmed cases of human infection and over 250,000 deaths in just a few short months [2]. It is known that the species specificity, host tropism, and transmission capacity of the virus are determined by the viral envelope spike (S) protein mediating the receptor-binding affinity to the host receptor. Therefore, to elucidate the pathogenesis of SARS-CoV-2 and develop vaccines and treatments for COVID-19, appropriate preclinical animal models that not only express human angiotensin-converting enzyme (ACE2), a functional receptor of SARS-CoV-2, but also can recapitulate the symptoms, is immediately required [3, 4]. Here, we describe the role of ACE2 in COVID-19 and the urgent need of translational research using human (h) ACE2 transgenic (Tg) mice, which are not only susceptible to the COVID-19 infection but, to varying degrees, also demonstrate the symptoms observed in human patients infected with this pandemic disease.

## Angiotensin-converting enzyme 2 (ACE2)

**Fig. 1:**
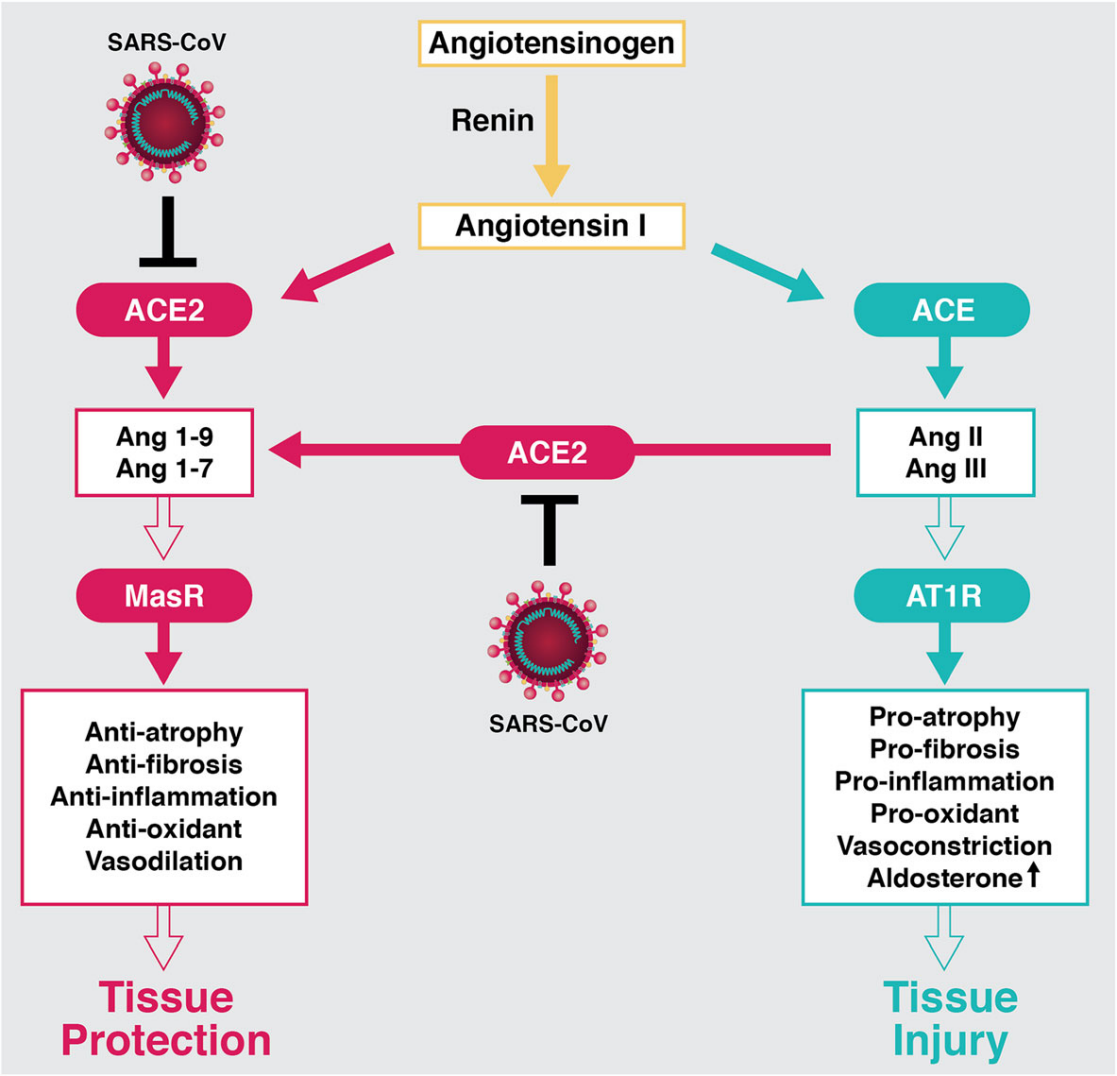
The role of the ACE2 and the adverse effect of SARS-CoV in the RAAS. Renin cleaves angiotensinogen into angiotensin I (Ang I), and the circulating Ang I is hydrolyzed to Ang II by ACE. Ang II activates the AT1R to lead the pro-atrophy, pro-fibrosis, pro-inflammation, pro-oxidant, vasoconstrictor response, and increase aldosterone synthesis. ACE2 directly hydrolyzes Ang I and Ang II to generate Ang1–9 and Ang1–7, respectively. Ang1–7 binds to the MasR which leads to the cellular signaling that opposite to the tissue injury effects of Ang II and does not stimulate aldosterone secretion. The SARS-CoV destroys the balance of RAAS by downregulating the ACE2 expression levels. Conclusively, the disproportion between AT1R and MasR axes in SARS-CoV infected patients contributes to the development of tissue injury and more severe inflammatory reactions

ACE2, known as ACE-related carboxypeptidase or angiotensin-converting enzyme homologue (ACEH), is a monocarboxypeptidase that is expressed in the lung, arteries, heart, kidney, brain, and intestines [5, 6], and is an essential component of the renin-angiotensin-aldosterone system (RAAS) [7]. The RAAS is a hormone system leading to the regulation of blood pressure, water balance, electrolytic homeostasis, vascular resistance, and heart remodeling [8]. The classical pathway of RAAS is initiated when circulating blood volume is decreased; the juxtaglomerular cells lining the afferent arterioles located proximal to the renal glomeruli recognize this change and secrete renin into blood circulation. Renin cleaves angiotensinogen into angiotensin I (Ang I) in the liver. The circulating Ang I is hydrolyzed to Ang II by ACE that is primarily located in the pulmonary and renal vessels. Ang II activates the angiotensin type I receptor (AT1R) to initiate a vasoconstrictor response and stimulate aldosterone synthesis in the adrenal gland. Then, both circulating blood volume and blood pressure are increased when aldosterone induces the renal tubes to initiate sodium and water retention [9, 10]. In RAAS, ACE2 directly hydrolyzes Ang II to yield Ang1–7 or converts Ang I to Ang1–9, eventually creating the vasodilator, Ang1–7, which is the most crucial product of ACE2. Ang1–7 binds to the G protein-coupled Mas receptor (MasR) which leads to the cellular signaling that, opposite to the vasoconstrictor effects of Ang II, does not stimulate aldosterone secretion (Fig. 1) [6, 9, 11, 12]. It is now recognized that the RAAS is far more complicated than the initial understanding of its classical function [13]. The imbalance between the levels of Ang I, Ang II, and Ang cleavage peptides (Ang1–9) leads to vasoconstriction and impairs vascular reactivity. As a key enzyme in controlling the balance of players in RAAS, ACE2 plays a critical role in cardio-renal and pulmonary disease [14, 15].

## The role of ACE2 as a receptor in the pathogenesis of SARS-CoV-2

ACE2 has been implicated as a functional cellular receptor for both SARS-CoV, the pathogen of SARS, which caused more than 8000 cases of infection and 774 deaths in 37 countries from 2002 to 2003 [16], and SARS-CoV-2 [17]. There have been abundant studies conducted to prove the essential role of ACE2 in the pathogenesis and severity of SARS-CoV [18–24], and leveraging this rich knowledge is highly beneficial and useful to elucidate the uses of ACE2 in the SARS-CoV-2 infection considering the similarity in both viral structure and pathogenesis between the two viruses [25]. Viral binding ability of SARS-CoV to ACE2 is known as the most crucial factor that supports the viral replication in the host and determines the infection efficiency in different species, including human, mice, rats, and palm civets [26–30]. Although the species responsible for transmission remains unclear [31, 32], several studies have shown that as SARS-CoV did, SARS-CoV-2 also originated from the bat and may also contain partial genes from another intermediate species, the pangolin (scale anteater) with high conservation in the genome which encode the spike proteins. The receptor-binding motif (RBM) in the receptor-binding domain (RBD) of SARS-CoV-2 is nearly identical to the one from the virus isolated in the pangolin [33–35]. By aligning the sequence of amino acids (AAs) in viral RBD to AAs in ACE2, five residues in hACE2, including K31, E35, D38, M82, and K353, were identified as the key AAs for interacting with viral RBM of both SARS-CoV and SARS-CoV-2 [36, 37]. Mainly, K31 and K353 were virus binding hot spots that play critical roles in the cross-species and human to human transmissions [36, 38, 39]. Recently, the ACE2 binding affinity to the RBD of both SARS-CoV and SARS-CoV-2 was evaluated in forty-two mammals based on hot spot AAs in the ACE2 of each of the species. The protein complex structure simulation study supported the possibility that pangolin ACE2 might have a better affinity to SARS-CoV-2 because the N82 of pangolin ACE2 could contact closer to the viral RBD than M82 of hACE2 [40].

The major clinical characteristics of SARS-CoV and SARS-CoV-2 patients are the deterioration of lung function and the apparent loss of lung repair capacity [41, 42]. ACE2 is expressed primarily in alveolar epithelial type II cells, which produce surfactant to prevent collapse of the alveoli in order to maintain normal gas exchange in the lung [17, 43]. The interaction between ACE2 and the RBD of the viral spike protein leads to endocytosis of virus particles through internalization with ACE2 and establishes SARS-CoV infection [44], leading to cell damage [45, 46]. Although the catalytic pocket of ACE2 was not blocked by binding to RBD, SARS-CoV infection notably downregulated the ACE2 expression at the transcriptional and posttranslational level [20–23]. This is validated by both in vitro and in vivo studies. Challenged with recombinant spike protein of SARS-CoV, cell culture downregulated ACE2 expression levels [47] and injection of SARS-CoV spike into mice decreased ACE2 expression levels, leading to a worsened lung injury (Fig. 1) [47, 48]. Furthermore, inflammation, which is excessively produced by infections of both SARS-CoV-2 [25] and SARS-CoV [46, 49–53], can suppress ACE2 transcription [54, 55], resulting in the RAAS activation. This activation contributes to the development of severe acute respiratory distress syndrome (ARDS) or acute lung injury, and more severe inflammatory reactions [48]. This suggests that direct viral interaction and the subsequent inflammation contribute to the ACE2 downregulation, thusly ACE2 potentially could serve to protect the lung from injury [56]. However, there are concerns about normalization or upregulation of ACE2 expression levels in patients because that would enable both a heightened level of infectivity of SARS-CoV-2 as well as clinical illness severity [57]. Indeed, the pathogenic events caused by these viral infections have been recognized as highly complex and not fully understood [58], so solely targeting the ACE2-mediated pathways alone may not be able to fully answer the questions regarding diversity of symptoms. Studies elucidating the pathogenesis and evaluating the efficacy of potential therapeutics and vaccines must be immediately conducted by utilizing the appropriate preclinical model.

## hACE2 Tg mouse models

Relevant animal models are essential in understanding the pathogenesis of both SARS [59] and COVID-19 [60]. Several animal models have been shown to be susceptible to SARS-CoV infection, such as ferrets, hamsters, mice, and non-human primates, which include macaques, African green monkeys, and marmosets [28, 45, 61–65]. These animals exhibited viral replication with a limited degree of histopathology and clinical illness, but none of them displayed consistent disease symptoms, an immunological response profile, or mortality [59]. The spike protein of SARS-CoV has a much higher binding affinity to hACE2 than to that of mice, rats, and other animal species, which correlates with the much lower level of permissiveness to this virus by these animals [24]. Therefore, some Tg mouse models expressing hACE2 were developed and used to elucidate the complex ACE2-mediated responses in SARS-CoV infection [59, 66]. These hACE2 Tg mice can provide significant findings to discover the pathogenesis of SARS-CoV-2 and support the development of COVID-19 therapeutics and vaccines.

### Human cytokeratin 18 (K18)-hACE2 Tg mouse models

**Fig. 2:**
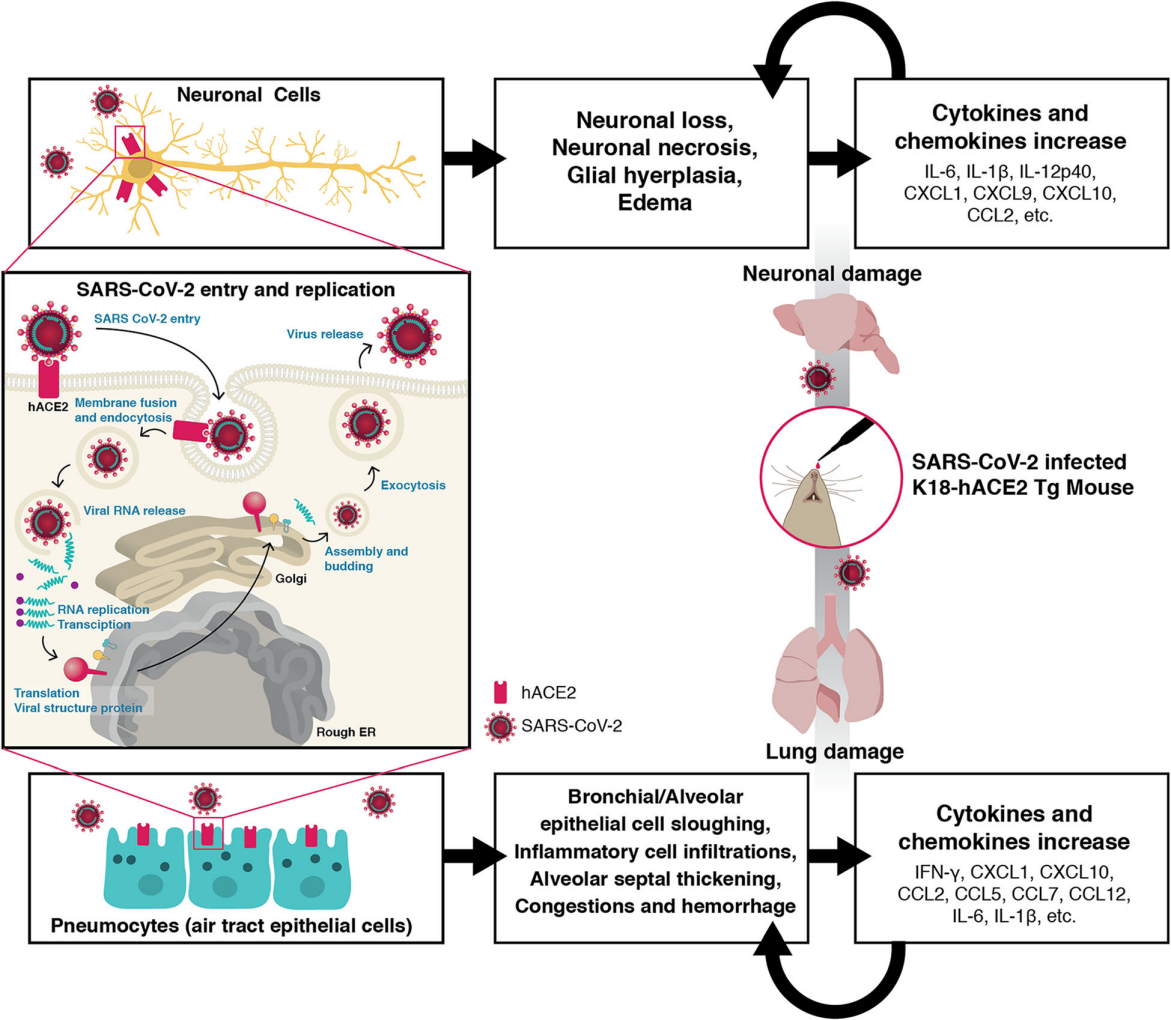
The potential pathogenic events in the SARS-CoV-2 infected K18-hACE2 mouse model. This diagram shows the pathogenic events that likely occur in SARS-CoV-2 infected hACE2 Tg mice using the examples of K18-hACE2 responses by SARS-CoV infection. K18-hACE2 Tg mice showed severe lung injury and neuronal damage in CNS by SARS-CoV infection, which were associated with the viral replication levels in each organ. The viral spike protein binds to the hACE2 expressed on neuronal cells or epithelial cells of the air tracks. The viral particles-hACE2 complex is internalized into cells. The virus replicates the viral RNA and generates the viral structure proteins to propagate themselves and infect other cells again. The cell damages, apoptosis, and infiltrated inflammatory cells by SARS-CoV-2 infection cause the tissue damage along with the cytokine and chemokine increases in the infected area, resulting in severe organ damages

K18-hACE2 Tg mice, also known as B6.Cg-Tg(K18-ACE2)2Prlmn/J, were developed by McCray et al*.* in 2006 [66, 67]. The purified DNA fragment containing the hACE2 coding sequence and 5′ and 3′ genomic regions of the human K18 gene, shown to be required for driving high-level epithelial cell-specific expression, was injected into pronuclei of zygotes from the intercross of (C57BL/6J × SJL/J) F_2_ parents to generate transgenic embryos (Table 1). The mice were then backcrossed onto a C57BL/6 background. The hACE2 mRNA expression was detected in several tissues, including the lung, liver, kidney, and colon, and a very low but measurable mRNA level of hACE2 was found in the brain [66]. Intranasal inoculation with SARS-CoV caused the development of rapidly fatal disease with the outcome correlated by the copy number and hACE2 mRNA level. The mice with the high hACE2 expression level (Tg lines 1 and 2) succumbed from day 3 to 5 post-infection (p.i.), whereas in the Tg line 3 which showed a lower hACE2 expression died 5 to 7 days p.i. Viral replication was found in the lungs of both K18-hACE2 Tg and non-Tg mice; however, the viral titers were lower and clearance much faster in the non-Tg mice. K18-hACE2 Tg mice began to lose weight by 3 to 5 days following SARS-CoV infection becoming lethargic with labored breathing, and all died within 7 days (Table 1) [66]. Similar to the patient’s symptoms, the lung was most obviously the organ majorly affected by SARS-CoV infection in K18-hACE2 Tg mice, showing significant inflammatory reactions (IFN-gamma, CXCL-1, CXCL-10, IL-6, IL-1beta, etc.) hemorrhage, epithelial cell damage, and congestion of alveolar septum (Fig. 2) [66, 67]. One of the more interesting findings regarding these mice was the heavy viral infection in the brain with increased inflammatory cytokines (CXCL-1, CXCL-10, IL-6, IL-1beta, etc.) (Fig. 2), postulated to be a major factor in the aspiration pneumonia observed in K18-hACE2 Tg mice and occasionally in infected patients as well [66]. In fact, there were several studies that detected virus in the brain of patients infected with SARS-CoV [46, 50, 72]. Some patients who survived this viral disease displayed the neurological/psychological sequelae that are presumed to be the side effects of either a corticosteroid therapy or a severe lung infection [72–75]. Further intensive investigation of SARS-CoV-induced neurological disease was a challenge due to the difficulty in obtaining infected brain tissues derived from patients [67]. Therefore, the K18-hACE2 Tg mouse was used to discover the pathogenic mechanism of SARS-CoV, including viral entry into the central nervous system (CNS), the spread of the neuronal infection, and the cause of lethality [67]. By detecting viral antigens in the different regions of the mouse brain and observing time-dependently, the neuronal infection of SARS-CoV was revealed to initiate from the olfactory bulb, spreading into the brain thoroughly 2 to 3 days after intranasal inoculation of the virus and induced neuronal loss [67]. The brain of the patient infected with SARS-CoV exhibited neuronal necrosis, glial hyperplasia, and edema while the viral infection mainly affected neurons [46, 50, 72], which is consistent with studies showing a distinguished neuronal tropism of SARS-CoV in infected K18-hACE2 Tg mice [59, 66]. Based on these SARS studies which utilized the K18-hACE2 Tg mice, some possible mechanisms including the high regional infection of the cardiorespiratory center in the medulla oblongata and the extreme inflammatory reactions that resulted in a “cytokine storm” were also suggested [67].

**Table 1:** The comparison of outcomes in each hACE2 Tg mouse model to SARS-CoV infection

	K18-hACE2 [66, 67]	AC70, AC22, and AC63 [59, 68]	HFH4-ACE2 [69]	Mouse ACE2 promoter-driven hACE2 Tg mice [70]
**Promoter**	Human K18 promoter	CAG promoter	Human HFH4 promoter	Mouse ACE2 promoter
**Parental mice of zygotes**	(C57BL/6J × SJL/J) F2	(C57BL/6J × C3H/HeJ) F1	(C3H × C57BL/6) F1	ICR
**Viral strains**	Urbani	Urbani	Urbani	PUMC01
**TCID50**^**a**^**of SARS-CoV**	1.6 × 10^4b^	AC70: 10^3^ AC22: 10^6^ AC63: 10^6^	7 × 10^4c^	10^5^
**Mortality (%)**	Line 1: 100 Line 2: 100 Line 3: 100	AC70: 100 AC22: 0 AC63: 0	100	0
**Survival days (p.i.)**	Line 1: 2–5 Line 2: 3–4 Line 3: 5–7	AC70: 4–8 AC22: n.a.^d^ AC63: n.a.	5–6	n.a.

^a^*TCID50* 50% tissue culture infective dose

^b^The viral dosage used in the study, 2.3 × 10^4^ plaque-forming units (PFU), was converted to the estimated TCID50 by the conversion TCID50 ≈ 0.7 PFU [71].

^c^The viral dosage used in the study, 10^5^ PFU, was converted to the estimated TCID50 by the conversion TCID50 ≈ 0.7 PFU [71].

^d^Not applicable

### AC70, AC22, and AC63 hACE2 Tg mouse models

In 2006, Tseng at. al, developed the hACE2 Tg mouse lineages (i.e., AC-12, AC-22, AC-50, AC-63, and AC-70) expressing hACE2 under the CAG promoter, a composite promoter consisting of the cytomegalovirus immediate early enhancer, the chicken β-actin promoter, rabbit globulin splicing, and polyadenylation sites to drive high levels of gene expression in eukaryotic expression vectors [59]. The plasmid containing hACE2 cDNA coding sequence was injected into pronuclei of zygotes from the (C57BL/6J × C3H/HeJ) F1 (Table 1). Either C56BL/6 or BALB/c mice were used for backcrossing. Each mouse line showed different expression levels of hACE2 mRNA. The AC70 Tg line, which is supremely susceptible to SARS-CoV infection, expressed a high level of hACE2 mRNA in overall organs (i.e., spleen, stomach, heart, muscle, brain, kidney, lung, intestine, and liver) and showed clinical illness, including lethargy, labored breathing, persistent weight loss leading to immobility, and 100% mortality within 4 to 8 days following infection (Table 1) [59, 68]. Among the tissues examined, the lung and brain were the primarily affected organs in the infected AC70 Tg mice and viral replication in the lung and brain showed notably different kinetics. While the viral titers reached the maximum level in the lung 1 to 2 days p.i. then gradually decreased, the viral titers in the brain were first detected on day 2, then rapidly increased on day 3 and remained at high levels until the death of the host. The inflammatory cytokine levels were correlated with the level of viral replication in the lung and brain. Following intraperitoneal injection which caused a low-level viremia in AC70 Tg mice, the virus was detected in the brain, but not in the lung, suggesting that the SARS-CoV could disseminate into the CNS by a mechanism independent from the one of lung infection [59]. Other Tg mice lines, AC22 and AC63, were used to investigate resistance to lethality following SARS-CoV infection as compared to AC70. Infected AC22 and AC63 Tg mice recovered even though the mice suffered from a severe clinical illness during the progression of the disease [59, 68]. Although the hACE2 expression levels in both the lung and brain are lower in AC22 than in AC70, the pattern of viral yield and the pulmonary pathologies in the lung following the SARS-CoV infection showed subtle dissimilarity between the two lines. However, both viral replication and inflammatory response in the brain of infected AC22 were significantly lower than those of AC70. Another finding in this comparison study showed the T cell loss in AC70 Tg mice was much more distinct than that in AC22 Tg mice, which was similar to the severity of T cell loss correlated with the poor outcome in the SARS-CoV and SARS-CoV-2 infected patients [52, 76–80].

### Hepatocyte nuclear factor-3/*forkhead homologue 4(*HFH4)-ACE2 Tg mouse models

HFH4-ACE2 Tg mice, also known as B6J.Cg-Tg (FOXJ1-ACE2)1Rba/Mmnc with airway-targeted overexpression of human *ACE2*, were generated by Menachery et al. in 2016. The expression cassette, consisting of HFH4 (*FOXJ1*) lung ciliated epithelial cell-specific promoter elements and the coding region of *ACE2* cDNA in a pTG1 vector, was injected into the pronuclei of zygotes from (C3H × C57BL/6) F1 (= C3B6F1) (Table 1). The founder mice were crossed to C3B6, and the hACE2 expression in each mouse was confirmed by PCR. The HFH4-ACE2 mice expressed hACE2 not only in the lung but also in the brain, liver, kidney, and gastrointestinal tract, indicating the broader organ distribution of HFH4-mediated expressions than expected initially. With SARS-CoV (Urbani strain) challenge, all HFH4-hACE2 mice rapidly lost more than 20% bodyweight and died within 4 to 5 days with robust viral replication in both the lung and brain found at the endpoint (Table 1). The anti-SARS human antibody 227.15 protected HFH4-hACE2 mice from death by SARS-CoV infection. These mice were used to evaluate the possibility of human infection and transmission of WIV1-CoV, which is a SARS-like virus identified by metagenomics studies and an isolated replication virus from the horseshoe bat known to be an animal reservoir of SARS-CoV [69]. In previous research, WIV1-CoV was revealed to have a broad species tropism and use ACE2 orthologs. Also, it could replicate at a low level in A549, a human lung cancer cell line [81]. Furthermore, the robust WIV1-CoV infection was confirmed in the well-differentiated primary human airway epithelial (HAE) cell, meaning that some SARS-like viruses which have not yet been found, as well as WIV1-CoV, might not need an intermediate species to infect humans. Therefore, it was critical to investigate the pathogenesis and observe symptoms of WIV1-CoV infection in animal models expressing hACE2. Compared to SARS-CoV infection in HFH4-hACE2 mice, WIV1-CoV infection showed attenuated symptoms, such as a lesser amount of weight loss with delayed death in limited mice as well as lower viral titer which contrasts the finding of a similar titer level following infection in primary HAE cell culture. This suggests that the WIV1-CoV spike protein can bind to hACE2, but additional adaptations will possibly be required to cause the epidemic disease [69]. This example demonstrates the additional use of hACE2 Tg mice for validation of potential epidemical viral strains, showing the beneficial impact of hACE2 Tg mice which can provide the knowledge needed for the prevention and management of future CoV outbreaks.

### Mouse ACE2 promotor-driven hACE2 Tg mouse models

The hACE2 Tg mouse, which had the hACE2 gene introduced into the mouse genome, was developed by Yang et al. in 2007 [69]. The hACE2 cDNA was inserted into the pEGFP-N1 plasmid and the hACE2 expression mediated under mouse ACE2 promotor, resulting in the likelihood of mimicking the human condition more closely. After the verification of hACE2 expression, the fragment containing the mouse ACE2 driving the hACE2 coding sequencing was injected into the pronuclei of the zygote from ICR mice (Table 1). The hACE2 expression was observed in the lung, heart, kidney, and intestinal tracts in this hACE2 Tg model. In contrast to the previous hACE2 Tg mice models, including K18-hACE2, A70, and HFH4-ACE2, although severe diffuse interstitial pneumonia and broad extrapulmonary organ damage were seen [69], similar to that observed in some patients infected SARS-CoV [82–84], these mice did not die by SARS-CoV infection (Table 1). The reason for this milder disease progression and recovery could be due to lower hACE2 expression levels. Since the death rate of SARS in 2003 was about 10% [70] and that of COVID-19 is about 7% [2], this more resistant hACE2 Tg mice, still with significant pathologic symptoms, is useful in understanding the pathogenesis of both SARS-CoV and SARS-CoV-2.

## The potential contributions of hACE2 Tg mouse models in the battle against COVID-19

### Development of vaccines

Several vaccine candidates for SARS-CoV were shown to induce neutralizing antibodies as well as being effective in protecting young mice or hamsters from the viral challenge [85–92]. The hACE2 Tg mouse, K18-hACE2, was used to test the protective ability of a recombinant SARS-CoV which is deleted in envelope (E) protein (rSARS-CoV-ΔE) or deleted in E and several group-specific protein genes (rSARS-CoV-Δ[E,6-9b]) against the SARS-CoV-mediated fatal respiratory disease. These rSARS-CoVs showed protective effects against challenge with SARS-CoV by inducing both anti-virus T cell and antibody responses and improved survival rate [93]. Recently, SARS-CoV-2 mRNA-1273, the inactivated recombinant-spike protein of SARS-CoV-2, was undergoing testing in clinical phase I, open-label, dose-ranging, clinical trial (NCT04283461) to mainly determine the safety and efficacy [57]. Since the virus can generate more subclasses, the efficacy, safety, host immunization, and potential to deter viral replication of SARS-CoV-2 should be evaluated in more vaccine candidates. To accomplish this desperate need, use of a hACE2 Tg mouse model will help to discover potential vaccines with desirable properties, and move toward the next phase of vaccine development.

### Evaluation of the potential therapeutics

Several strong therapeutic candidates, most were previously used for the treatment of viral disease [94–97], have been rapidly applied to clinical trials for COVID-19 based on their anti-viral effects validated in cultured cells [96, 97]. For instance, remdesivir (GS-5734), which has been recognized as a broad-spectrum anti-viral drug against RNA viruses [97], such as SARS and MERS-CoV [98], was shown to inhibit SARS-CoV-2 viral infection in a human liver cancer cell line (Huh-7) [97]. Remdesivir was recently approved by the FDA for emergency use in severe COVID-19 patients [99] and will further be evaluated for efficacy when given to patients earlier in their disease course. Currently, there are eight clinical trials in progress in the USA (NCT04315948, NCT04292730, NCT04280705, NCT04302766), China (NCT04252664, NCT04257656), and France (NCT04314817, NCT04315948) to assess the expanded treatment approaches and potential combinational treatments of remdesivir and to get the approval from the FDA. Considering the complicated and hidden pathogenic mechanisms and evolution of SARS-CoV-2, an in vivo study should be simultaneously and immediately conducted to provide more comprehensive data that can predict the therapeutic and adverse responses in SARS-CoV-2 infected patients. The hACE2 Tg mice may provide highly beneficial data, particularly that associated with the severity of COVID-19, because clinical illness or mortality cannot be investigated using cultured cells or non-transgenic mice. Moreover, hACE2 Tg mice can facilitate developing and evaluating the potential therapeutic strategy targeting the hACE2-mediated pathogenic pathways. The soluble recombinant human ACE2 (rhACE2) has been attracting attention as a competitive interceptor that can bind to RBD of the SARS-CoV or SARS-CoV-2 before the viral RBDs attach to the full-length hACE2 which is bound to the cell membrane [100]. Several in vitro studies have shown that the soluble form of ACE2 blocked the SARS-CoV replication in Vero-E6 cells [101, 102] and the extracellular domain of human ACE2 fused to human IgG1-Fc could neutralize the SARS-CoV-2 [103]. If an appropriate form of rhACE2 can be provided, it would be a new therapeutic compound used to inhibit SARS-CoV-2 binding to ACE2 and to diminish viral infection and viral replication in the host [100]. However, the beneficial effect of rhACE2 has not yet been tested in animal studies or human clinical studies; therefore, hACE2 Tg mice would be a useful animal model that can expedite the process of developing this new approach.

### Elucidation of the pathogenesis

Subsequent to migration and mutation SARS-CoV-2 has been evolving into subclasses. Tang et al. reported that two major lineages (L-type and S-type) of SARS-COV-2 co-exist by phylogenetic analysis of 103 genomes with SARS-CoV-2. The S-type was indicated as the ancestral subclass, and the L-type was characterized to have a higher frequency than S-type [104]. The co-infection of these two subclasses was observed in one patient (USA_2020/01/21.a, GISAID ID: EPI_ISL_404253), but it was not clear whether the co-infection increased the severity or not [104]. More recently, three subclasses (i.e., A, B, and C subclasses) were identified based on the distinguishable AA changes by character-based phylogenetic network analysis of 160 SARS-CoV-2 genomes [105]. Understanding the pathogenesis of each viral subclass consisting of special residues determining host affinity or disease severity is important for developing vaccines and treatment. As described before, the clinical symptoms in hACE2 Tg mice following SARS-CoV infection were quite similar to that in patients. hACE2 Tg mice will potentially provide significant knowledge about viral dissemination and pathogenic impact of SARS-CoV-2 subclasses in infected hosts. Also, it will lead to essential knowledge that supports further transgenic animal models for enhancing or investigating the permissiveness and infection of SARS-CoV-2 [106].

### Validation of the risk factors associated with the severe symptoms in COVID-19

In a previous study using mouse models, aged mice have shown more severe pathological lesions and a higher mortality rate than young mice following SARS-CoV infection, and young mice required more mutations and passages than aged mice to produce the mouse-adapted SARS-CoV strains [107–112]. Age is known as one of the factors that determine disease severity in SARS and COVID-19 [57, 113]. Therefore, investigating the pathologic severity of SARS-CoV-2 associated with age in young and old hACE2 Tg mice is immediately required to understand the difference of symptoms in infected patients and establish a disease-control strategy. Also, in COVID-19, most of the patients with lethal symptoms were revealed to have pre-existing medical comorbidities, such as pulmonary disease, chronic kidney disease, diabetes mellitus, obesity (body mass index ≥ 30), cardiovascular disease, and hypertension [114–119]. There have been various animal models developed for investigating these diseases including inbred strains of mice with genetic manipulations that predispose them to disease [120–123]. Other manipulations and environmental exposures can be used to study the effects of comorbidities such as cigarette smoke- or drug (i.e., bleomycin)-induced pulmonary disease [124, 125], paraquat-induced acute renal failure [126], repeated low-dose cisplatin treatment-induced chronic renal failure [127], streptozotocin-developed type I diabetes mellitus [128], and high fat/sucrose dites-induced insulin resistance, which is closely linked with type II diabetes melilitus, obesity, and cardiovascular disease [129, 130]. The chemical/diet-induced disease methods can be applied to hACE2 Tg mouse models before infecting the SARS-CoV-2 to elucidate the association between comorbidity and the symptoms or pathogenesis of COVID-19. Moreover, the further development of breeding together Tg mice that develop comorbidities and express hACE2 will facilitate the discovery of the impact and mechanism of each comorbidity on the severity of symptoms in COVID-19. This knowledge will help us to be ready, prevent, and protect people from an undesirable pandemic disaster like COVID-19.

## Conclusion

SARS-CoV-2 has a higher binding affinity to hACE2 than to that of other species, and the recent Cryo-Electron microscopy studies demonstrate that it showed even a higher affinity than SARS-CoV [131]. The transgenic mouse model that expresses hACE2 has been proven to mimic the symptoms of human disease by SARS infection. We have comprehensively reviewed the currently established hACE2 Tg mouse models and the studies utilizing these mice. Also, arguments were presented that show the urgent demand for investigations of COVID-19 treatments and prevention significantly require the use of hACE2 Tg mice. In conclusion, along with the efforts in all medical and scientific fields, the hACE2 Tg mice are a critical translational animal model that would greatly facilitate the deciphering of this pandemic disease and development of therapeutics and vaccines against COVID-19.

## Data Availability

Not applicable.

## Abbreviations

AAs: Amino acids
ACE: Angiotensin-converting enzyme
ACE2: Angiotensin-converting enzyme 2
ACEH: Angiotensin-converting enzyme homologue
Ang I: Angiotensin I
Ang II: Angiotensin II
Ang1–7: Angiotensin 1–7
Ang1–9: Angiotensin 1–9
ARDS: Acute respiratory distress syndrome
AT1R: Angiotensin type I receptor
CNS: Central nervous system
CoV: Coronavirus
COVID-19: Coronavirus disease 2019
HAE: Human airway epithelial
HFH4: Hepatocyte nuclear factor-3/forkhead homologue 4
K18: Cytokeratin 18
MasR: Mas receptor
p.i.: Post-infection
PFU: Plaque-forming units
RAAS: Renin-angiotensin-aldosterone system
RBD: Receptor-binding domain
RBM: Receptor-binding motif
rSARS-CoV-ΔE: Recombinant SARS-CoV envelope (E) protein
S protein: Spike protein
SARS: Severe acute respiratory syndrome
SARS-CoV: Severe acute respiratory syndrome-associated coronavirus
SARS-CoV-2: Severe acute respiratory syndrome-associated coronavirus-2
TCID50: 50% tissue culture infective dose
Tg: Transgenic
